# Validation of a Mucosal IgA Assay for SARS-CoV-2

**DOI:** 10.3390/microorganisms14051154

**Published:** 2026-05-20

**Authors:** Mingzhu Zhu, Edmond Massuda, Shane Cloney-Clark, Urvashi Patel, Anand Parekh, Andrew Gorinson, Andrew Klindworth, Ali Ahmadi, Miranda R. Cai, Chijioke Bennett, Raj Kalkeri, Joyce S. Plested

**Affiliations:** 1 Clinical Immunology, Novavax, Gaithersburg, MD 20878, USA; mzhu@novavax.com (M.Z.);; 2 Biostatistics, Novavax, Gaithersburg, MD 20878, USA; 3 Clinical Development, Novavax, Gaithersburg, MD 20878, USA

**Keywords:** COVID-19, immunogenicity, SARS-CoV-2, assay validation, correlate of protection, Omicron XBB.1.5

## Abstract

Mucosal immunity, including antibodies like immunoglobulin A (IgA), function as the body’s first line of defense in the respiratory tract, particularly against viruses. An anti-rS protein IgA enzyme-linked immunosorbent assay (ELISA) was developed using the Omicron XBB.1.5 subvariant of SARS-CoV-2 and was validated to demonstrate the suitability of the method for testing saliva from SARS-CoV-2 vaccine clinical trials. This assay successfully met acceptance criteria for inter-/intra-assay precision, specificity, selectivity, linearity, lower/upper limits of quantitation, and assay robustness. The IgA in saliva was stable for up to 7 freeze/thaw cycles, for up to 48 h at 24 °C, up to 7 days at 4 °C, up to 3 weeks at −20 °C, and up to 1 year at −80 °C. After validation using Omicron XBB.1.5 rS protein, cross-reactivity was demonstrated with the SARS-CoV-2 variant JN.1. This validated IgA assay can be a valuable tool to assess mucosal IgA levels in SARS-CoV-2 clinical trials.

## 1. Introduction

Mucosal immunity functions as the body’s first line of defense in the respiratory tract against foreign substances, especially viruses that are spread via respiratory droplets from mucosal secretions [1,2,3,4]. Immunoglobulin A (IgA) is a class of antibodies, including IgA1 and IgA2, that are predominant in mucosal and nasal secretions [1,2,3,4]. Similarly to IgG, IgA antibodies neutralize the activity of a range of pathogens, including viruses [1,4]. After infection, IgA levels tend to peak earlier than IgG levels [3,5,6], and high mucosal IgA levels are an indicator of protection against COVID-19; by contrast, low mucosal IgA levels are linked to potential susceptibility to SARS-CoV-2 infection [7].

Due to these rapid responses and the role of IgA in the defense against SARS-CoV-2 [8,9,10], IgA measurement could be a useful indicator to assess mucosal immunity as a mechanism of protection from viral infection. An anti-rS (recombinant spike protein) IgA enzyme-linked immunosorbent assay (ELISA) was developed using the Omicron XBB.1.5 subvariant as an assessment of vaccine effectiveness, measuring mucosal IgA levels before and after vaccination. This assay was validated for XBB.1.5, and evaluated with JN.1, to demonstrate the suitability of the method for testing saliva IgA from SARS-CoV-2 vaccine clinical trials.

## 2. Materials and Methods

### 2.1. Assay Procedure

**Figure 1:**
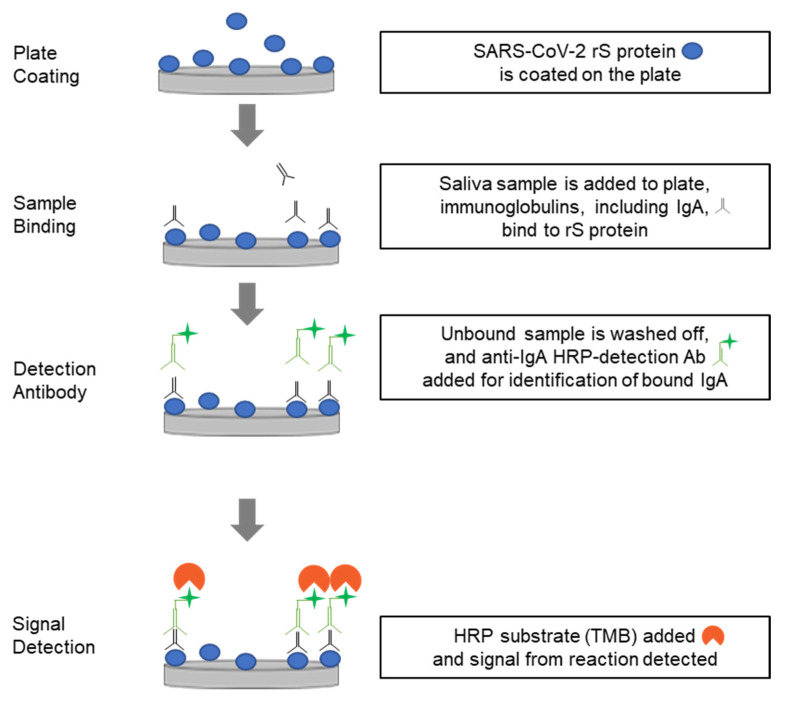
Procedure for performing the anti-rS IgA detection assay. Optical density of the signal is read at 450 nm and average IgA concentration reported as ng/mL. Ab, antibody; IgA, immunoglobulin A; HRP, horse radish peroxidase; rS, recombinant spike; TMB, 3,3′,5,5′-tetramethylbenzidine.

An ELISA-based procedure was developed to detect IgA antibodies against SARS-CoV-2 Omicron XBB.1.5 in saliva samples. Assays to detect JN.1 and the ancestral (Wuhan) strain IgA were also developed. The 96-well assay was conducted as shown in Figure 1.

SARS-CoV-2 rS protein (produced at Novavax, Inc., Gaithersburg, MD, USA) was immobilized onto the surface of 96-well microtiter plate wells (Nunc Maxisorp plate; Thermo Fisher Scientific, Waltham, MA, USA) by direct absorption for 16 to 48 h at 2–8 °C. Coated plates were washed with phosphate-buffered saline with Tween 20 (PBST) and blocked (blocking buffer, Thermo Fisher Scientific, Waltham, MA, USA, Cat# 37538) for 1–1.5 h. Reference standards (human IgA1 and IgA2 anti-SARS-CoV-2 rS protein antibodies; AcroBiosystems Cat # S1N-M164 and SPD-M521, Newark, DE, USA) and assay quality controls (human IgA1 and IgA2 anti-SARS-CoV-2 rS protein antibodies; from AcroBiosystems Cat # S1N-M164 and SPD-M521) were diluted in pooled saliva from commercial (BioIVT, Westbury, NY; Precision for Medicine, Norton, MA, USA; Medix Biochemica [pre–COVID-19], Maryland Heights, MO, USA) and clinical (rS protein from participants of the phase 3 2019nCoV-314 study/NCT05973006 [11]; see Table 1 in Zhu et al. (2025) [12]) sources. Clinical saliva samples were collected using the Salivette^®^ method (application of an absorbent roll in the mouth). Participants rinsed their mouth with water prior to collection and samples were isolated ≥60 min after a meal, teeth brushing, or ingestion of oral medication (see [12] for details). Pre–COVI-19 commercial saliva samples were negative when screened for endogenous anti-rS IgA binding.

Saliva dilutions were added to the rS protein-coated wells and any specific antibodies were allowed to complex with the immobilized rS antigen (2 h of incubation). After washing the plates with PBST, antibodies bound to rS proteins immobilized to the surface were then detected using a secondary goat anti-human IgA antibody conjugated with horseradish peroxidase (HRP) (Invitrogen, Carlsbad, CA, USA, Cat# A18787) incubated for 1 h at room temperature. After a final wash step, the 3,3′,5,5′-tetramethylbenzidine substrate (TMB, Sigma, Cat# T00440-1L, St. Louis, MO, USA) was added and the reaction was stopped after 20 min by TMB stop solution (Scytek Laboratories, Cat# TSB999, Logan, UT, USA). When detection reagents are in excess, the optical density (OD) of the chromogenic substrate is proportional to the quantity of anti-rS IgA in the saliva samples, which was measured at 450 nm on an ELISA plate reader. The results were analyzed by SoftMax^®^ Pro software version 7.1.1 (Molecular Devices, LLC; San Jose, CA, USA). The total IgA antibodies in test saliva samples were quantitated against a reference standard on the same plate.

The saliva samples were diluted 1:10 in assay buffer (1% milk in phosphate-buffered saline), followed by 1:2 serial dilutions for a total of 11 dilutions (1:10 to 1:10,240). The anti-rS protein IgA level at all dilution points was calculated using a reference standard based on OD values at each dilution point. Since the samples were serially diluted and anti-rS protein IgA antibodies were measured at multiple dilution points (interpolated concentration and multiplied by dilution factor), the resulting values with a coefficient of variation (CV) ≤20% were averaged and reported as the anti-rS protein IgA concentration (ng/mL).

### 2.2. Assay Validation

Assay performance was evaluated for precision, linearity, selectivity, specificity, sensitivity, stability, and robustness. Precision was defined as the closeness of agreement (degree of scatter; CV) between a series of 12 measurements of the same sample. Intra-assay precision (repeatability) and inter-assay precision (intermediate precision), as well as total variation, which is the combined intra-assay and inter-assay variation, were evaluated by testing 18 saliva samples (15 pooled human saliva clinical trial samples [2019nCoV-314] and three quality control [QC] samples) that covered the high- (HQC), mid- (MQC), and low- (LQC) ranges of the assay. Clinical samples with similar IgA concentrations were pooled to generate QC samples with sufficient volume for use in validation assays [13]. The HQC sample had 125 ng/nL IgA added and the MQC and LQC samples were naturally positive for IgA to SARS-CoV-2 S protein. Each sample was tested twice within an assay run, in six different runs by two analysts on three different days, generating 12 results. The intra- and inter-assay precision was estimated by calculating percent geometric coefficient of variation (%GCV) based on the variance component analysis, using analyst and day as random effects and the samples as a fixed effect. Variance component analysis was performed on the natural log-transformed anti-rS protein IgA results. Acceptance criteria were defined as ≥80% of samples having intra-assay GCV ≤ 20% and ≥80% of samples having inter-assay GCV ≤ 20%.

Linearity evaluated proportionality of test results to the amount of IgA in the sample. Linearity was assessed in a pooled clinical saliva sample with high-concentration IgA and tested in five assay runs. Linearity was based on a relative bias ≤ 20% of expected anti-rS IgA for samples with IgA greater than the lower limit of quantitation (LLOQ) and within 25% for samples at/near the LLOQ; a GCV ≤ 20% for all samples; and *R*^2^ ≥ 0.95 and a regression line slope 95% CI between 0.8 and 1.25. Sensitivity was based on linearity data used to assess the lowest anti-rS protein IgA level that can be determined with acceptable precision (≤25% GCV) and relative accuracy (% relative bias within 25% of the expected value), defined as the assay LLOQ.

Selectivity assessed anti-rS IgA measurement in pre-pandemic saliva, which should be below the LLOQ to demonstrate minimal matrix interference. There were 40 pre–COVID-19 samples (Medix Biochemica, St. Louis, MO, USA) collected between 2016 and 2019. Post–COVID-19 samples (n > 350) were from baseline (pre-dose) of the 2019nCoV-314 trial (see Table 1 in Zhu et al. (2025) [12]).

Specificity assesssed the ability of the assay to measure and differentiate specific IgA in the presence of other components. Saliva samples were incubated with homologous protein (XBB.1.5 spike), related protein (SARS-CoV-2 [Wuhan] spike), or unrelated protein (respiratory syncytial virus [RSV]). The assay tested for specificity with the following criteria: ≥50% inhibition of detection by homologous protein for at least 80% of the samples and no significant (<20%) inhibition of detection with unrelated protein. Percent inhibition was calculated as:

(1) $$\%Inhibition\;=\;[100\;-\;\frac{Results\;with\;protein\;incubation}{Results\;without\;protein\;incubation}\;\times \;100]$$

Stability was assessed by evaluating the sample performance within the expected handling and storage limits. Stability was tested for commerical samples that underwent up to seven freeze/thaw cycles and for varied durations at RT, 4 °C, and −20 °C, and for storage of clinical samples at −80 °C.

Robustness was evaluated based on the reproducibility of the assay for various hours of plate coating and various sample and detection antibody incubation times, with the goal of ≥80% of samples being with 20% of baseline values for acceptance.

## 3. Results

### 3.1. SARS-CoV-2 XBB.1.5 Mucosal IgA Assay Validation

#### 3.1.1. Assay Quality Control Samples

**Figure 2:**
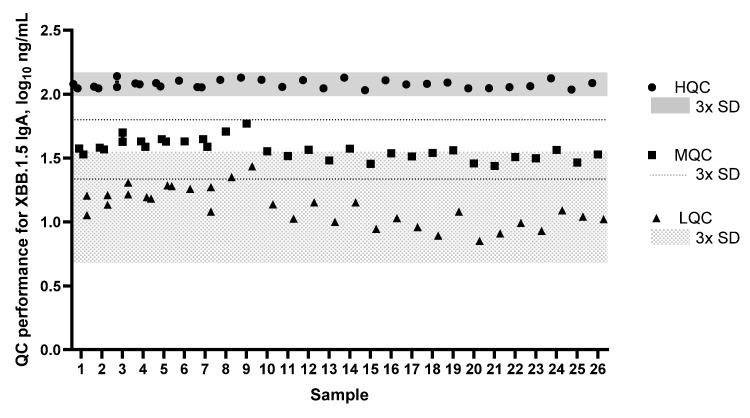
QC performance for XBB.1.5 IgA for high QC (HQC), mid QC (MQC), and low QC (LQC). Shaded areas (HQC and LQC) and dotted lines (MQC) represent ±3 × SD of the average for each QC sample set. IgA, immunoglobulin A; QC, quality control.

All QC sample performance met the targeted criteria, with the hierarchy of HQC > MQC > LQC (Figure 2). The upper and lower limits of QCs were calculated as ±3 SD. The HQC was within 96.8 and 147.7 ng/mL, the MQC was within 21.6 and 63.1 ng/mL, and the LQC was within 5.0 (LLOQ) and 35.4 ng/mL.

#### 3.1.2. Assay Precision

Assay precision was tested among 15 pooled clinical saliva samples and three QC samples. The overall inter-assay and intra-assay %GCV were 10.8% and 7.0%, respectively (Table 1). For both intra- and inter-assay precision, 17/18 (94.4%) samples met the criteria of ≤20% GCV, successfully demonstrating assay precision.

#### 3.1.3. Assay Linearity

**Figure 3:**
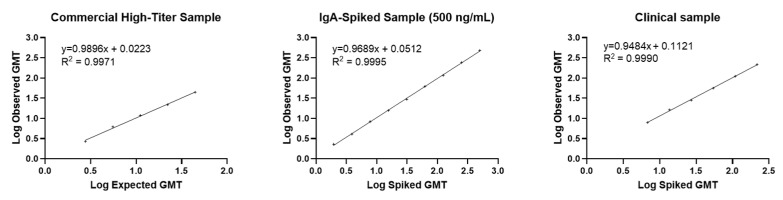
Assay linearity for salivary IgA with SARS-CoV-2 XBB.1.5 assay. GMT, geometric mean titer; IgA, immunoglobulin A.

Linearity was assessed in two commercially available saliva samples (one low-titer that was IgA_1_ and IgA_2_-spiked at 500 ng/mL) and a pooled clinical sample of high-concentration IgA serially diluted to a point presumed < LLOQ. The expected LLOQ was assigned to be 5.0 ng/mL. The ULOQ was assigned to be 500 ng/mL, which was the highest concentration where precision was confirmed. Linearity was successfully demonstrated, with *R*^2^ values of 0.9971 and 0.9990 for the commercial samples and 0.9990 for the clinical sample (Figure 3). The lowest expected IgA level (6.8 ng/mL) above the LLOQ was at the sixth dilution factor of 32. Relative bias within this range of dilution factors was acceptable for five of the first six dilution factors (range: 0–20.7).

#### 3.1.4. Assay Selectivity

**Figure 4:**
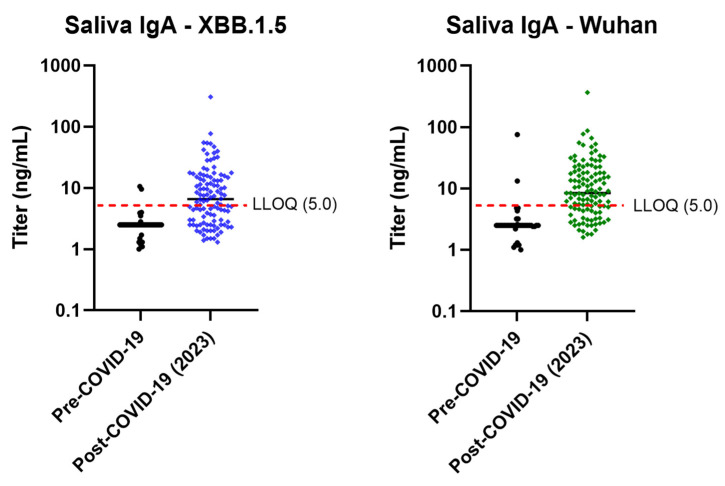
Assay selectivity for mucosal IgA assay, comparing titers in pre– and post–COVID-19 samples. IgA, immunoglobulin A; LLOQ, lower limit of quantitation.

Selectivity of the assay, based on 40 pre–COVID-19 IgA saliva samples, was successfully demonstrated for both XBB.1.5 and Wuhan strains (Figure 4). If selective, SARS-CoV-2 IgA would not be identified in pre–COVID-19 samples (isolated from 2016 to 2019). LLOQs for 38/40 (95.0%) and 37/39 (94.9%) pre–COVID-19 XBB.1.5 and Wuhan samples, respectively, were <5 ng/mL.

#### 3.1.5. Assay Specificity

Specificity of the IgA assay to XBB.1.5 was successully demonstrated in four clinical saliva samples with detectable IgA (Table 2) [13]. Antibody detection inhibition was observed for Omicron XBB.1.5 rS protein at 1.0 μg/mL and 2.0 µg/mL. Pre-incubation with an irrelevant protein (RSV F) did not show significant inhibition (<20% inhibition) across all four saliva samples.

#### 3.1.6. Sample Stability

**Figure 5:**
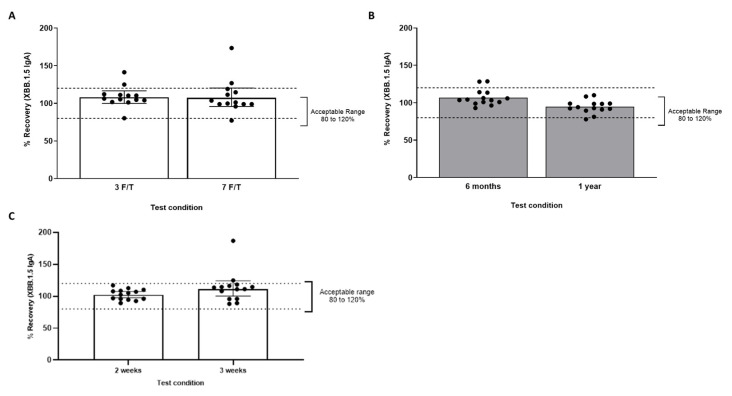
Temperature and freeze/thaw stability of saliva samples for the IgA assay with storage at −80 °C. (**A**) Recovery after 3 or 7 F/T cycles for 13 commercial saliva samples. (**B**) Recovery after storage of 14 clinical saliva samples for 6 months or 1 year. (**C**) Recovery after storage of 14 saliva samples at −20 ± 10 °C for 2 and 3 weeks. F/T, freeze thaw cycles; IgA, immunoglobulin A.

The saliva sample freeze/thaw (Figure 5A) and storage (Figure 5B) stability were successfully demonstrated in commercial and clinical samples, respectively. Among 14 commercial saliva samples, 13 (92.9%) had 70–130% recovery after storage at room temperature (24 °C) for 48 h, and 12 (85.7%) had 80–120% recovery after refrigeration (2–8 °C) for 7 days. Of samples stored at −20 °C for 2 weeks and 3 weeks, 100% and 85.7%, respectively, met acceptance criteria for stability (Figure 5C; 80–120% recovery; see Materials and Methods in Section 2). After storage of commercial samples at −80 °C and three freeze/thaw cycles, 85.7% of samples were within 80–120% of baseline values. Following seven freeze/thaw cycles, 78.6% (11 of 14) remained within acceptance limits. One sample was near the LLOQ (5) and exhibited a recovery of 126.8% with no evidence of loss of antibody activity; this result was considered acceptable. Overall, the data met the acceptance criteria for freeze/thaw stability.

#### 3.1.7. Assay Robustness

**Figure 6:**
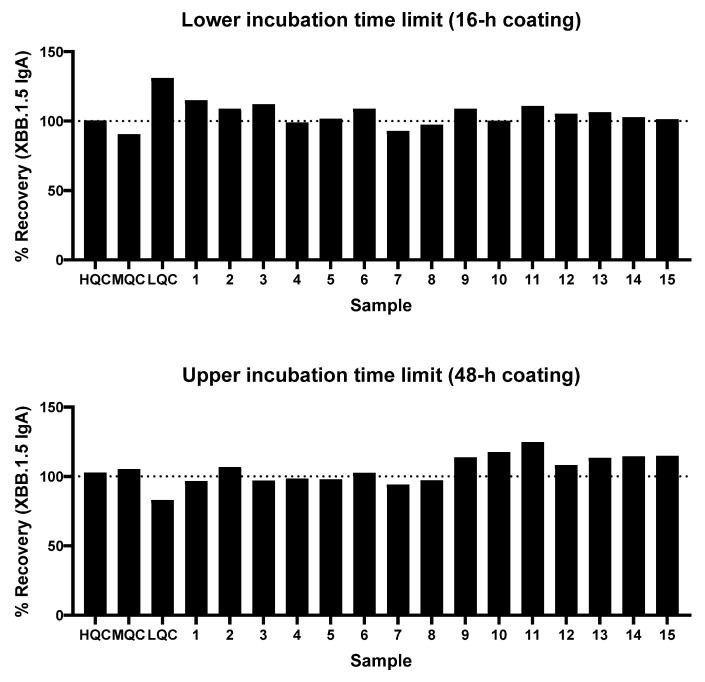
IgA assay robustness based on incubation time limits and plate coating time. HQC, high QC; LQC, low QC; MQC, mid QC; QC, quality control.

The robustness of the assay was assessed with a total of 18 clinical saliva samples (3 QC and 15 pooled clinical samples) across multiple parameters, including plate coating time and lower and upper limits of sample incubation time for each step of the assay (Figure 6). The ideal range of time of XBB.1.5 rS protein coating was found to be between 16 h and 48 h. At both the lower incubation time limits and 16-h coating and upper incubation time limit and 48-h coating, 17 out of 18 samples (94.4%) had recovery between 80% and 120%.

### 3.2. SARS-CoV-2 Mucosal IgA Assay Variant Crossreactivity

**Figure 7:**
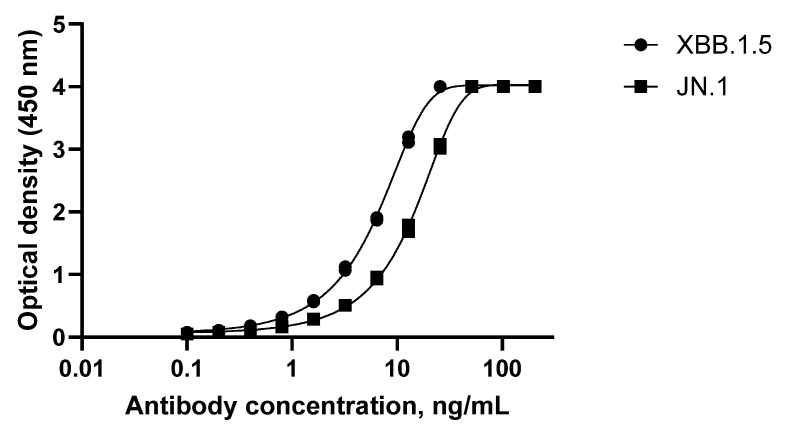
XBB.1.5 IgA assay cross-reactivity for SARS-CoV-2 variant JN.1. A dilution series of 12 antibody concentrations were analyzed.

The original assay was developed using Omicron XBB.1.5 and demonstrated cross-reactivity to the JN.1 variant strain (Figure 7). Both antibodies in the reference IgA1 and IgA2 bound to JN.1, although somewhat less than XBB.1.5.

## 4. Discussion

The results from this assay validation demonstrate that this method for measuring the anti-Omicron XBB.1.5 S protein IgA antibody in human saliva of vaccinated participants is precise, linear, and specific. Stability testing demonstrated that all short-term and longer-term storage conditions were acceptable, including 4 °C for 7 days, −20 °C for 2 and 3 weeks, −80 °C for 6 months and 1 year, and up to seven freeze/thaws from −80 °C. SARS-CoV-2 IgA assays were also developed for Wuhan and JN.1. Both reference IgA antibodies bind to JN.1, but somewhat less than XBB.1.5. As previously reported, cross-reactivity of salivary IgA to JN.1 was observed in clinical samples from participants of the 2019nCoV-314 study [12].

Mucosal IgA has emerged as a significant factor in the context of COVID-19 vaccines, particularly for its role in preventing SARS-CoV-2 replication at the site of infection and limiting transmission [8,9]. Reduced viral transmission and infection by elevated secretory IgA in the upper respiratory tract suggests that mucosal IgA may be a reliable marker of vaccination-induced mucosal immunity and a critical component of next-generation vaccine development [14,15].

The importance of mucosal immunity emphasizes the need for standardized methods to assess related antibodies and their roles as correlates of protection. Collaborative studies have explored the mucosal immune response to SARS-CoV-2 infection and vaccination, revealing that saliva can be used to measure mucosal immunity [16]. These findings suggest that mucosal IgA may potentially serve as a correlate of protection against breakthrough infection; however, its induction is limited by current vaccine formulations and intramuscular administration.

Evidence from non-human primate studies indicates that vaccines delivered intranasally or via aerosol elicit durable airway IgA responses, which correlate with protection in the upper respiratory tract [17]. Unlike intramuscular mRNA vaccines, mucosal vaccines also induce spike-specific B cells in the lungs, highlighting their potential for a broader and more localized immune response. Notably, the Matrix-M^®^ adjuvant used in the Novavax COVID-19 vaccine has demonstrated significant benefits for mucosal immunity, predominantly triggering immune responses localized to tissues rather than systemic spillover [18]. This localized immune activation is crucial for mucosal immunity, as it enhances the body’s ability to respond to infections at the primary entry points of respiratory pathogens. Future COVID-19 vaccine strategies should focus on enhancing mucosal immunity to achieve comprehensive protection against SARS-CoV-2; however, validated IgA assays are needed to assess mucosal immunity in these clinical investigations. Current recommendations for testing for SARS-CoV-2 infection do not include IgA testing, likely because of limitations in detection, as noted in descriptions of other assays [19,20].

Selectivity of the assay described here was demonstrated based on pre– and post–COVID-19 IgA saliva samples. These results are in line with previous reports of IgA assay validations demonstrating a strong IgA response after natural infection [21,22]. Specificity in this assay was high, with antibody detection inhibition by Omicron XBB.1.5 rS protein demonstrated in four clinical saliva samples. Other available IgA assays have demonstrated specificity ranging from 64.3% to 84.4% [23].

There was no correlation observed between salivary IgA and other serum markers (IgG, PNT) for the 2019nCoV-314 study (for Omicron XBB.1.5, Spearman correlation, *R*^2^ < 0.2 saliva IgA vs. PNT, *R*^2^ < 0.2 saliva IgA vs. serum IgG), due to the potentially inherent differences between the salivary IgA (secreted) vs. serum IgG (different compartments) [24]. These data highlight the importance of measuring the salivary IgA, as it is unique and different compared to other serum markers.

One limitation to this assay is that it does not distinguish between secretory IgA and systemically produced IgA; however, it does recognize both forms. Notably, this distinction will be a more relevant factor with intranasal vaccines that induce secretory IgA than those delivered via intramuscular injection and primarily induce serum IgG, IgA, and IgM [25]. Additionally, the clinical samples that were used for Wuhan and XBB.1.5 validation are from study 2019nCoV-314, which are all from adolescent participants who received the Novavax COVID-19 vaccine containing rS to XBB.1.5 or a bivalent formulation to XBB.1.5/Wuhan by intramuscular route of vaccination.

## 5. Conclusions

These insights highlight the growing recognition of mucosal IgA as a correlate of protection, especially for local mucosal immune protection, paving the way for innovative vaccine approaches targeting mucosal immunity. The IgA assay described here can be a valuable tool to assess mucosal IgA levels in saliva samples from SARS-CoV-2 clinical trials of novel and developing vaccines.

## Figures and Tables

**Table 1:** Precision summary for mucosal IgA assay with SARS-CoV-2 XBB.1.5.

Sample	N ^1^	GMC (ng/mL)	Inter-Assay %GCV	Intra-Assay %GCV	Total %GCV
Overall ^2^	N/A	N/A	10.8	7.0	12.9
HQC ^3^	12	127.8	7.2	6.3	9.6
MQC	12	32.7	13.9	10.7	17.6
LQC	12	8.3	26.4	4.9	26.9
1	12	43.0	10.6	3.7	11.2
2	12	63.0	7.7	4.2	8.8
3	12	48.5	10.7	4.8	11.8
4	12	17.3	12.2	3.3	12.7
5	12	23.8	12.2	3.4	12.7
6	12	35.9	9.6	4.0	10.4
7	11	16.5	16.2	5.6	17.1
8	11	11.1	14.4	6.6	15.9
9	11	26.4	8.6	5.9	10.5
10	11	9.8	11.5	3.9	12.2
11	12	11.6	9.1	6.4	11.2
12	12	27.9	12.0	5.2	13.0
13	12	24.2	9.6	5.1	10.9
14	12	52.1	8.5	3.2	9.1
15	12	13.2	6.7	5.9	8.9

^1^ Number of values used in the calculation. ^2^ The overall assay precision is general assay precision, calculated by considering all samples listed. ^3^ IgA-spiked sample. GCV, geometric coefficient of variation; GMC, geometric mean concentration; HQC, high QC; LQC, low QC; MQC, mid QC; N/A, not available; QC, quality control.

**Table 2:** Specificity summary for mucosal IgA assay.

Sample	Assay Buffer	Omicron XBB.1.5 rS Protein 2.0 μg/mL		Omicron XBB.1.5 rS Protein 1.0 μg/mL	
	Ab (ng/mL)	Ab (ng/mL)	% Inhibition	Ab (ng/mL)	% Inhibition
Clinical 1	23.6	5.9	75.0	5.7	75.8
Clinical 2	20.8	<5	88.0	<5	88.0
Clinical 3	17.9	<5	86.0	<5	86.0
Clinical 4	13.7	<5	81.8	<5	81.8
**Sample**	**Assay Buffer**	**RSV F Protein 1.0 μg/mL**			
Clinical 1	23.6	22.0	6.8		
Clinical 2	20.8	19.8	4.8		
Clinical 3	17.9	16.1	10.1		
Clinical 4	13.7	12.5	8.8		

Note: Values of 2.5 (half of LLOQ) were used for calculation purposes of sample titers <LLOQ. Ab, antibody; IgA, immunoglobulin A; rS, recombinant spike protein; RSV, respiratory syncytial virus.

## Data Availability

The data presented in this study are available on request from the corresponding author due to proprietary patient and sample information.
